# Chronos: Stress Makes the Clock Tick

**DOI:** 10.1371/journal.pbio.1001838

**Published:** 2014-04-15

**Authors:** Richard Robinson

**Affiliations:** Freelance Science Writer, Sherborn, Massachusetts, United States of America

[Fig pbio-1001838-g001]There is a clock inside every mammalian cell whose complexity exceeds that of any Swiss watch. Made of genes and proteins linked in mutually controlling feedback loops, the cellular clock regulates both itself and multiple circadian processes, including secretion, protein synthesis, and response to stress. The whole-body clock that governs our sleep–wake cycle and that is entrained by exposure to light is built upon the cellular clock.

**Figure pbio-1001838-g001:**
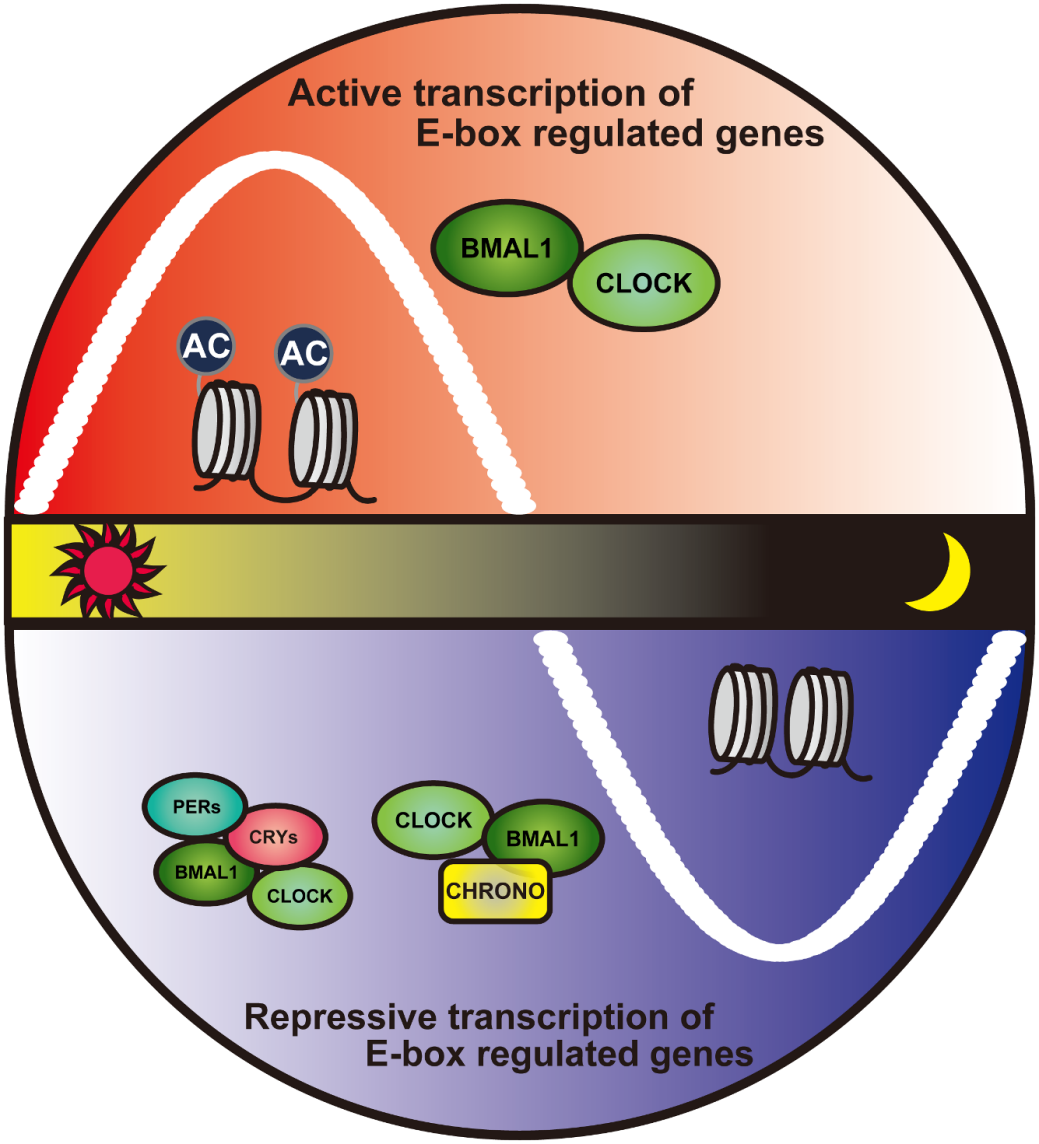
CHRONO negatively regulates circadian oscillations by directly interacting with the clock transcription factor BMAL1.

Beginning in the early 1990s, the pieces of the clock have gradually become known, with most researchers believing that most of the major components have now been identified. The parts list takes another major step towards completion in this issue of *PLOS Biology*, as two independent groups describe the discovery of an important new clock gene.

Ron Anafi, John Hogenesch, and colleagues began their search for new clock genes by defining five features of known ones: an endogenous expression cycle with an approximately 24-hour period; loss of expression causing an effect on circadian behavioral rhythm; interaction with other known clock components; expression in most tissues; and sequence conservation between flies and vertebrates. None of these was considered an absolute requirement, they reasoned, but the more features a gene demonstrated the more likely it was to be involved in the clock.

With that in mind, the authors surveyed multiple mammalian cell types with a suite of tools to measure temporal changes in transcription, effect of RNAi knockdown, protein–protein interactions, and sequence homology of genes across the genome. Scores were assigned for each feature, and cumulative scores were used to prioritize genes for further investigation. Reassuringly, among the top 20 genes were 10 canonical clock genes, and several more appeared in the top 50. Among the top previously unidentified genes was *Gm129*.

That same gene emerged in the work of Akihiro Goriki, Toru Takumi, and colleagues, who performed a genome-wide chromatin immunoprecipitation analysis for genes that were the target of BMAL1, a core clock component that binds to many clock genes, regulating their transcription. Both groups rename *Gm129* “*Chrono*” for either “computationally highlighted” or “ChIP-derived” repressor of network oscillator.

The CHRONO protein interacted with BMAL1 and several other known clock proteins, with a phase opposite that of BMAL1. Knocking out *Chrono* led to an increase in the behavioural circadian period of about 25 minutes, about the same effect as knocking out any of a half dozen other core clock components. By selectively knocking out *Chrono* in mouse hypothalamus (the site of whole-body clock entrainment), Goriki et al. found that loss of the protein altered behavioral rhythms of the organism as a whole.

Goriki et al. also found that CHRONO interacted with histone deacetylase, in so doing helping to regulate multiple other genes by repressing their translation. The group also showed that organism-wide stress responses were regulated by CHRONO, through its interaction with the glucocorticoid receptor, repressing the receptor's function in a circadian fashion.

The existence of *Chrono* was predicted by persistence of circadian behavior after knocking out multiple other clock genes; its discovery now explains this observation, and should quickly help determine whether there are other major clock components still to be found. Its influence on stress responses and behavioral rhythms will likely be a major focus of future investigation, given their central importance to human health.

**Anafi RC, Lee Y, Sato TK, Venkataraman A, Ramanathan C, et al. (2014) Machine Learning Helps Identify CHRONO as a Circadian Clock Component.** doi:10.1371/journal.pbio.1001840

**Goriki A, Hatanaka F, Myung J, Kim JK, Yoritaka T, et al. (2014) A Novel Protein, CHRONO, Functions as a Core Component of the Mammalian Circadian Clock.** doi:10.1371/journal.pbio.1001839

